# Pica/Pagophagia-Associated Hyponatremia: Patient Presenting With Seizure

**DOI:** 10.7759/cureus.9330

**Published:** 2020-07-21

**Authors:** Genanew Bedanie, Alay Tikue, Thanita Thongtan, Mohamed Zitun, Kenneth Nugent

**Affiliations:** 1 Internal Medicine, Texas Tech University Health Sciences Center, Lubbock, USA; 2 Internal Medicine/Pulmonary and Critical Care Medicine, Texas Tech University Health Sciences Center, Lubbock, USA

**Keywords:** pica, pagophagia, iron deficiency anemia, hyponatremia, seizures

## Abstract

Pica is an unusual condition in which patients crave and chew substances with no nutritional value. Ice pica (pagophagia) is commonly seen in patient with iron deficiency. People chew ice cubes or add ice to their drinks to cool or refresh themselves, and they may not consider this as an abnormal behavior. Excessive ice chewing/eating can have significant health risks, including electrolyte abnormalities and metabolic disorders. We report a patient admitted to our hospital with severe hyponatremia and seizures due to iron deficiency-associated pagophagia. Ice pica leading to hyponatremia and seizure is not commonly seen in clinical practice. It was a challenging case and the patient was seen and investigated at outpatient clinic for polyuria, though an underlying cause was not identified. Patients may be secretive or reluctant to mention their pica habit. Studies have also shown that most physicians are unaware of pica and most cases can be easily missed.

## Introduction

Pica is described as an unusual condition in which patients crave and chew substances (such as ice, clay, soil, dirt, raw rice, or paper) that have no nutritive value [1, 2]. The term pica originates from the Latin word for magpie (Picave), a bird that is famed for its unusual eating behaviors, mainly it is known to eat almost anything. Pica was referenced by the Greeks and Romans in 13th-century but was not addressed in medical texts until 1563. ​Pagophagia (craving and chewing ice) is the excessive consumption of ice cubes or iced drinks; it is often associated with iron deficiency with or without anemia [2-4]. Less commonly, other nutritional disorders may cause craving and chewing ice. ​The reasons why these patients crave ice is unclear, but some studies have indicated that chewing ice might increase alertness in people with iron deficiency anemia. Pagophagia can have significant health risks, including electrolyte abnormalities and metabolic disorders. We report a case of a patient admitted to medical intensive care unit (MICU) due to severe hyponatremia and seizure, who was found to have iron deficiency-associated pagophagia as the underlying cause. Ice pica leading to hyponatremia and seizures is rare in clinical practice, and to our knowledge from literature review, prevalence is unknown. Studies have indicated most physicians are unaware of pica and most cases are missed [5, 6].

## Case presentation

A 54-year-old Caucasian woman was transferred to our MICU from an outside hospital due to altered mental status, seizures, and hyponatremia. ​On presentation to the outside hospital, she was weak and had a generalized tonic clonic seizure. Her serum sodium was as low as 120 millimoles/litre (mmol/L), serum osmolality 242 milliosmoles/kilogram (mOsm/kg), urine sodium 9 mmol/L and urine osmolality of 70 mOsm/kg. Prior to that hospital admission, the patient was complaining of excessive and frequent urination for several months. She was investigated for possible causes of polyuria by her primary care physician and finally referred to an endocrinologist. Her endocrinologist did not find the exact cause for her symptoms, and empirically the patient was started on desmopressin for polyuria. ​Her outpatient osmolality was always 260-280 mOsm/kg, she also had mild hyponatremia (133-136 mmol/L), urine sodium 14 mmol/L and urine osmolality of 110 mOsm/kg. ​Based on her outpatient laboratory results, diabetes insipidus seemed unlikely.

Psychogenic polydipsia was considered and desmopressin was discontinued at outside hospital, but the patient stated she was not drinking too much water and her water intake was 3-4 Liter/day. ​She was placed on normal saline, her serum osmolality and sodium were gradually corrected, and the patient was transferred to our medical center for high level of care. ​In our hospital, she was admitted to the MICU with possible psychogenic polydipsia, her laboratory test revealed serum sodium 128 mmol/L, serum osmolality 254 mOsm/kg, urine sodium 11 mmol/L and urine osmolality of 100 mOsm/kg. Upon further evaluation and lab reviews for anemia, the patient was asked if she was eating too much ice. She stated she was craving and chewing multiple bags of ice, which was unusual for her. The patient also mentioned she had abnormal uterine bleeding for which she was treated by a gynecologist. She had no history of similar craving and chewing of ice in the past and denied any current stress or psychiatric illness. On physical examination, the patient was alert and oriented; vital signs were normal. She had mild pale conjunctiva and non-icteric sclera. Chest was clear with normal heart sounds, no murmur or gallop. She had no abdominal tenderness, palpable organomegaly, or any neurologic deficits.

Her laboratory tests revealed a hemoglobin of 9.2 grams/deciliter and mean corpuscular volume (MCV) of 75 femtolitres.​ Iron deficiency with pica was considered, and an iron panel revealed: iron level 9 micrograms/deciliter (normal value 60-160 micrograms/deciliter), transferrin saturation 2% (normal value 20-50%), iron binding capacity 377 micrograms/deciliter (normal value 250-460 micrograms/deciliter), and ferritin level 4.8 nanograms/milliliter (normal value 15-200 nanograms/milliliter). Vitamin B12, folate, cortisol, and thyroid-stimulating hormone (TSH) were all normal. She had normal renal function and no anion gap. Computed tomography of head and electroencephalogram were normal. The patient was given parenteral iron infusion for five days with oral iron supplementation. Craving for ice improved significantly while the patient was in hospital following iron infusion. The patient was stable, had no more seizures, and was discharged with oral iron supplementation. She was seen on three-month follow-up and she stated craving for ice had resolved. Lab work revealed hemoglobin of 12.8 grams/deciliter, serum sodium 142 mmol/L and serum osmolality of 285 mOsm/kg.

## Discussion

Pica remains a mysterious and fascinating occurrence, and many physicians miss this diagnosis. ​Pagophagia, which is craving and chewing ice, is often associated with iron deficiency with or without anemia [1, 5, 6]. Interestingly, pica sufferers with low iron levels did not crave or eat items that were naturally high in iron [6]. It is commonly seen in pregnant women and in the pre-adolescent age group [4]. Iron absorption can also be reduced in the presence of non-nutritive substances. Pica is strongly associated with iron deficiency and is believed to be a symptom of deficiency rather than its cause [4, 7, 8]. In a few cases, pagophagia has been in association with compulsive behavior or depressive disorders. These patients use pagophagia as a coping mechanism to deal with their psychological stressors [9]. The prevalence of pica is not studied well, but published data from iron deficient pregnant women revealed that it ranges between 8-65%. According to the study in Latin America by Lopez et al., prevalence of pica was 23-44% in iron deficient pregnant women [2]. The diagnosis can be missed unless it is specifically asked. This under estimation is probably related to health professionals' unawareness and/or lack of knowledge about pica [2, 5, 6].

A study with 81 patients with iron deficiency anemia showed that revealed iron therapy can cure the pagophagia before the hemoglobin level recovers [10]. A case-control study in France from April 19, 1999 to April 18, 2000 involved 158 anemic patients admitted to a hospital. Non-food substances, mainly clay and ice, were ingested regularly by 35 patients (44%) with iron-deficiency anemia and by seven (9%) in the control group (P < 0.0001), but the prevalence of food craving did not differ significantly between the groups. A case series of three patients revealed resolution of pagophagia following iron therapy in patients who had severe iron deficiency anemia with a low serum ferritin level. These patients were eating many bags and trays of ice per day [7].

Studies have shown that many physicians are unaware of the pica symptoms [5, 6]. In addition, patients may be secretive or reluctant to mention their pica habit. This can make diagnosis difficult, and it can be missed unless the physician specifically addresses this possibility. Thus, pica can have significant health risks, including electrolyte abnormalities and metabolic disorders, secondary to a delayed diagnosis. A case series reported by Khan and Tisman showed all three of their patients with iron deficiency-related pica were missed by their primary care physician and academic supervisors [6]. It is recommended to evaluate patient with pica for iron deficiency and its causes. Iron therapy can cure the pagophagia earlier than hemoglobin recovery [10, 11]. If the cause of pica is an emotional or developmental disorder, cognitive ​behavioral therapy may be helpful [9].

## Conclusions

Continuous craving for ice may be a sign of an underlying condition that requires medical attention, but most patients are unaware of that and may not tell their physician. Our patient presented with seizure from hyponatremia due to iron deficiency-associated pagophagia. Her hyponatremia was also likely exacerbated by desmopressin use. This kind of presentation is rarely seen in clinical practice, and can be challenging for physicians to consider pica. Cases can be easily missed and misdiagnosed unless specifically addressed. We recommend physicians consider pica and ask specific questions if patient is chewing too much ice or not.

## References

[1] Coltman CA Jr (1969). Pagophagia and iron lack. Nutr Rev.

[2] Lopez LB, Ortega Soler CR, de Portela ML (2004). Pica during pregnancy: a frequently underestimated problem (Article in Spanish). Arch Latinoam Nutr.

[3] Parry-Jones B (1992). Pagophagia, or compulsive ice consumption: a historical perspective. Psychol Med.

[4] Borgna-Pignatti C, Zanella S (2016). Pica as a manifestation of iron deficiency. Expert Rev Hematol.

[5] Rose EA, Porcerelli JH, Neale AV (2000). Pica: common but commonly missed. J Am Board Fam Pract.

[6] Khan Y, Tisman G (2010). Pica in iron deficiency: a case series. J Med Case Rep.

[7] Osman YM, Wali YA, Osman OM (2005). Craving for ice and iron-deficiency anemia: a case series from Oman. Pediatr Hematol Oncol.

[8] Kettaneh A, Eclache V, Fain O (2005). Pica and food craving in patients with iron-deficiency anemia: a case-control study in France. Am J Med.

[9] Mehra A, Sharma N, Grover S (2018). Pagophagia in a female with recurrent depressive disorder: a case report with review of literature (Article in Turkish). Turk Psikiyatri Derg.

[10] Uchida T, Kawati Y (2014). Pagophagia in iron deficiency anemia (Article in Japanese). Rinsho Ketsueki.

[11] Reynolds RD, Binder HJ, Miller MB, Chang WW, Horan S (1968). Pagophagia and iron deficiency anemia. Ann Intern Med.

